# 5-Chloro-3-ethyl­sulfinyl-2-(3-fluoro­phen­yl)-1-benzo­furan

**DOI:** 10.1107/S1600536813018151

**Published:** 2013-07-06

**Authors:** Hong Dae Choi, Pil Ja Seo, Uk Lee

**Affiliations:** a Department of Chemistry, Dongeui University, San 24 Kaya-dong, Busanjin-gu, Busan 614-714, Republic of Korea; b Department of Chemistry, Pukyong National University, 599-1 Daeyeon 3-dong, Nam-gu, Busan 608-737, Republic of Korea

## Abstract

The asymmetric unit of the title compound, C_16_H_12_ClFO_2_S, contains two independent mol­ecules in which the benzo­furan ring systems are essentially planar, with r.m.s. deviations of 0.007 (1) and 0.013 (1) Å. In the crystal, mol­ecules are linked by weak C—H⋯O hydrogen bonds into chains extending along the *b* axis. These chains are further packed into stacks along the *c* -axis by S⋯O contacts [3.1898 (11) and 3.1361 (11) Å] involving the sulfinyl groups. In both 3-fluoro­phenyl rings, the F atom is disordered over two positions, with site-occupancy factors of 0.921 (2) and 0.079 (2).

## Related literature

For background information and the crystal structures of related compounds, see: Choi *et al.* (2010*a* ▶,*b* ▶). For details of sulfin­yl–sulfinyl inter­actions, see: Choi *et al.* (2008 ▶). For a review of carbon­yl–carbonyl inter­actions, see: Allen *et al.* (1998 ▶).
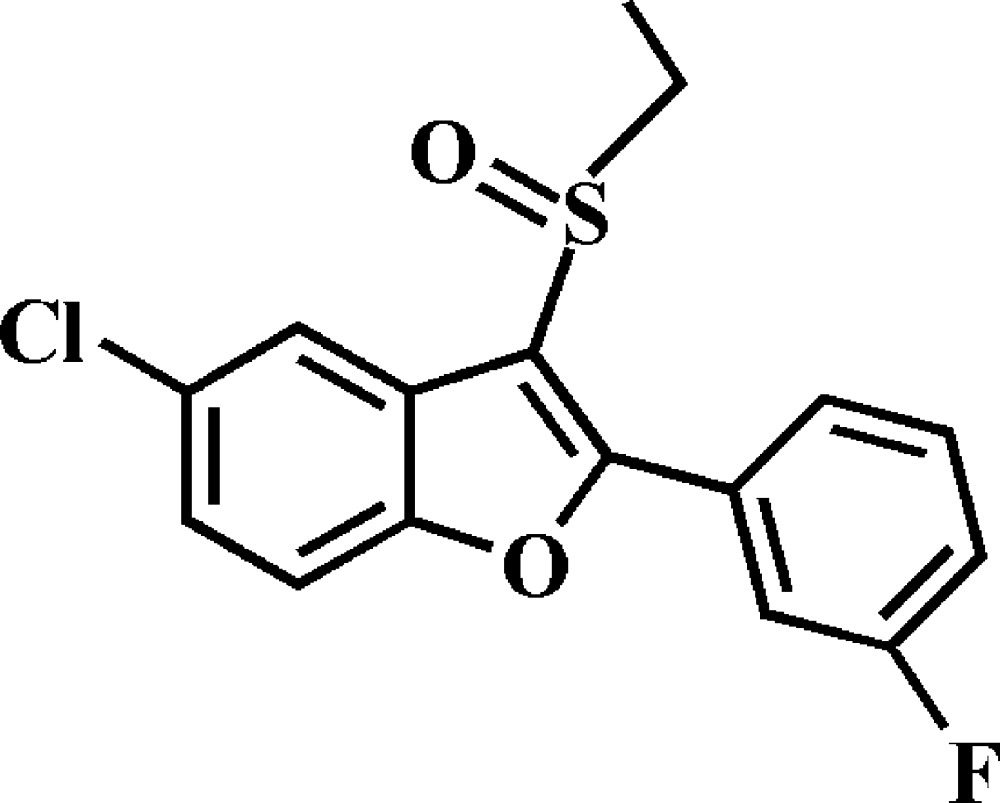


## Experimental

### 

#### Crystal data

C_16_H_12_ClFO_2_S
*M* *_r_* = 322.77
Triclinic, 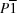

*a* = 9.5538 (5) Å
*b* = 11.2638 (5) Å
*c* = 13.4079 (6) Å
α = 98.739 (2)°
β = 93.733 (3)°
γ = 98.627 (2)°
*V* = 1404.25 (12) Å^3^
*Z* = 4
Mo *K*α radiation
μ = 0.43 mm^−1^
*T* = 173 K
0.39 × 0.30 × 0.13 mm

#### Data collection

Bruker SMART APEXII CCD diffractometer
Absorption correction: multi-scan (*SADABS*; Bruker, 2009 ▶) *T*_min_ = 0.625, *T*_max_ = 0.746
24991 measured reflections
6456 independent reflections
5012 reflections with *I* > 2σ(*I*)
*R*_int_ = 0.039

#### Refinement

*R*[*F* ^2^ > 2σ(*F* ^2^)] = 0.038
*wR*(*F* ^2^) = 0.102
*S* = 1.06
6456 reflections
390 parameters
16 restraints
H-atom parameters constrained
Δρ_max_ = 0.35 e Å^−3^
Δρ_min_ = −0.38 e Å^−3^

### 

Data collection: *APEX2* (Bruker, 2009 ▶); cell refinement: *SAINT* (Bruker, 2009 ▶); data reduction: *SAINT*; program(s) used to solve structure: *SHELXS97* (Sheldrick, 2008 ▶); program(s) used to refine structure: *SHELXL97* (Sheldrick, 2008 ▶); molecular graphics: *ORTEP-3 for Windows* (Farrugia, 2012 ▶) and *DIAMOND* (Brandenburg, 1998 ▶); software used to prepare material for publication: *SHELXL97*).

## Supplementary Material

Crystal structure: contains datablock(s) I. DOI: 10.1107/S1600536813018151/fj2636sup1.cif

Structure factors: contains datablock(s) I. DOI: 10.1107/S1600536813018151/fj2636Isup2.hkl

Supplementary material file. DOI: 10.1107/S1600536813018151/fj2636Isup3.cml

Additional supplementary materials:  crystallographic information; 3D view; checkCIF report

## Figures and Tables

**Table 1:** Hydrogen-bond geometry (Å, °)

*D*—H⋯*A*	*D*—H	H⋯*A*	*D*⋯*A*	*D*—H⋯*A*
C15—H15*B*⋯O4	0.99	2.28	3.233 (2)	160
C31—H31*B*⋯O2^i^	0.99	2.26	3.211 (2)	160

Symmetry code: (i) 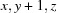.

**Fig. 1.**
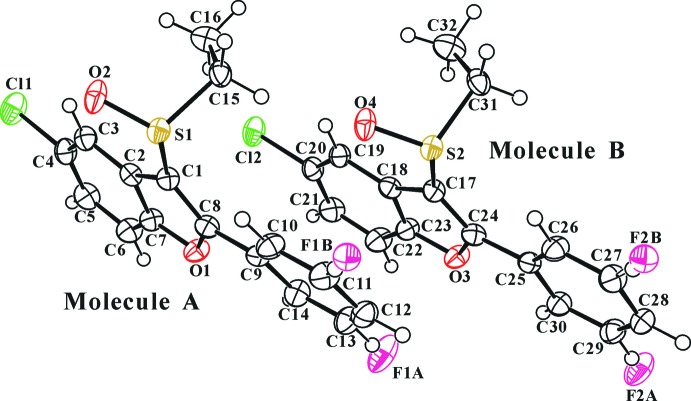
The molecular structure of the title compound with the atom numbering scheme. Displacement ellipsoids are drawn at the 50% probability level. H atoms are presented as small spheres of arbitrary radius. The F atom of the 3-fluorophenyl ring is disordered over two positions with site occupancy factors, from refinement of 0.921 (2) (part A) and 0.079 (2) (part B).

**Fig. 2.**
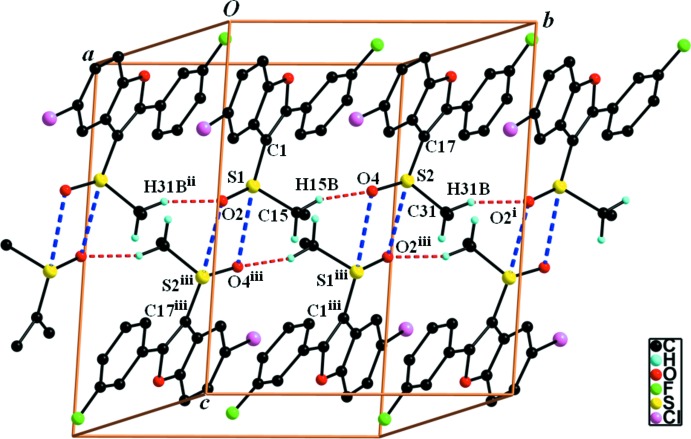
A view of the C—H···O and S···O interactions (dotted lines) in the crystal structure of the title compound. H atoms non-participating in hydrogen-bonding and disordered part B atoms were omitted for clarity. [Symmetry codes: (i) x, y + 1, z; (ii) x, y - 1, z; (iii) - x + 1, - y + 1, - z + 1.]

## supplementary crystallographic information

### Comment

As a part of our continuing study of 5-chloro-3-ethylsulfinyl-1-benzofuran derivatives containing 4-fluorophenyl (Choi *et al.*, 2010*a*) and 4-iodophenyl (Choi *et al.*, 2010*b*) substituents in 2-postion, we report herein the crystal structure of the title compound which crystallizes with two symmetrically independent molecules, A & B, in the asymmetric unit.

In the title molecule (Fig. 1), the benzofuran unit is essentially planar, with a mean deviation of 0.007 (1) and 0.013 (1) Å, for A and B molecule, respectively, from the least-squares plane defined by the nine constituent atoms. In the 3-fluorophenyl rings of both molecules, the F atoms are disordered over two positions with site-occupancy factors, from refinement of 0.921 (2) (part A) and 0.079 (2) (part B). The dihedral angles between the 3-fluorophenyl ring and the mean plane of the benzofuran ring system are 15.35 (8)° in the molecule A and 5.62 (9)° in the molecule B, respectively. In the crystal packing (Fig. 2), molecules are connected by weak C—H···O hydrogen bonds (Table 1) into chains extending along the *b-axis* direction.

In the crystal packing (Fig. 2), these chains are further packed into stacks along the *c-axis* by a sulfinyl–sulfinyl interaction (Choi *et al.*, 2008) interpreted as similar to a type-Il carbonyl–carbonyl interaction (Allen *et al.*, 1998), with S1···O4^iii^ and S2···O2^iii^ distances of 3.1898 (11) and 3.1361 (11) Å (symmetry code iii: - *x* + 1,- *y* + 1,- *z* + 1).

### Experimental

3-Chloroperoxybenzoic acid (77%, 269 mg, 1.2 mmol) was added in small portions to a stirred solution of 5-chloro-3-ethylsulfanyl-2-(3-fluorophenyl)-1-benzofuran (337 mg, 1.1 mmol) in dichloromethane (40 mL) at 273 K. After being stirred at room temperature for 5h, the mixture was washed with saturated sodium bicarbonate solution and the organic layer was separated, dried over magnesium sulfate, filtered and concentrated at reduced pressure. The residue was purified by column chromatography (hexane–ethyl acetate, 2:1 v/v) to afford the title compound as a colorless solid [yield 67%, m.p. 390–391 K; *R*_f_ = 0.59 (hexane–ethyl acetate, 24:1 v/v)]. Single crystals suitable for X-ray diffraction were prepared by slow evaporation of a solution of the title compound in acetone at room temperature.

### Refinement

All H atoms were positioned geometrically and refined using a riding model, with C—H = 0.95 Å for aryl, 0.99 Å for methylene and 0.98Å for methyl H atoms, respectively. *U*_iso_(H) = 1.2*U*_eq_(C) for aryl and methylene, and 1.5*U*_eq_(C) for methyl H atoms. The positions of methyl hydrogens were optimized rotationally. The F1 and F2 atoms of the 3-fluorophenyl rings are disordered over two positions with site occupancy factors, from refinement of 0.921 (2) (part A) and 0.079 (2) (part B). For the proper treatment of H-atoms, carbon atoms C11 and C13 (molecule A), and C27 and C29 (molecule B) were divided in two parts with equalized coordinates and thermal parameters. The distance of equivalent C–F pairs were restrained to 1.330 (5) Å using command DFIX, and displacement ellipsoids of F1 and F2 sets were restrained to 0.01 using command ISOR.

### Crystal data

**Table:**

C_16_H_12_ClFO_2_S	*Z* = 4
*M**_r_* = 322.77	*F*(000) = 664
Triclinic, *P*1	*D*_x_ = 1.527 Mg m^−^^3^
Hall symbol: -P 1	Melting point = 390–391 K
*a* = 9.5538 (5) Å	Mo *K*α radiation, λ = 0.71073 Å
*b* = 11.2638 (5) Å	Cell parameters from 7946 reflections
*c* = 13.4079 (6) Å	θ = 2.5–27.5°
α = 98.739 (2)°	µ = 0.43 mm^−^^1^
β = 93.733 (3)°	*T* = 173 K
γ = 98.627 (2)°	Block, colourless
*V* = 1404.25 (12) Å^3^	0.39 × 0.30 × 0.13 mm

### Data collection

**Table:**

Bruker SMART APEXII CCD diffractometer	6456 independent reflections
Radiation source: rotating anode	5012 reflections with *I* > 2σ(*I*)
Graphite multilayer monochromator	*R*_int_ = 0.039
Detector resolution: 10.0 pixels mm^-1^	θ_max_ = 27.5°, θ_min_ = 1.5°
φ and ω scans	*h* = −12→12
Absorption correction: multi-scan (*SADABS*; Bruker, 2009)	*k* = −14→14
*T*_min_ = 0.625, *T*_max_ = 0.746	*l* = −17→17
24991 measured reflections	

### Refinement

**Table:**

Refinement on *F*^2^	Primary atom site location: structure-invariant direct methods
Least-squares matrix: full	Secondary atom site location: difference Fourier map
*R*[*F*^2^ > 2σ(*F*^2^)] = 0.038	Hydrogen site location: difference Fourier map
*wR*(*F*^2^) = 0.102	H-atom parameters constrained
*S* = 1.06	*w* = 1/[σ^2^(*F*_o_^2^) + (0.0489*P*)^2^ + 0.2168*P*] where *P* = (*F*_o_^2^ + 2*F*_c_^2^)/3
6456 reflections	(Δ/σ)_max_ < 0.001
390 parameters	Δρ_max_ = 0.35 e Å^−^^3^
16 restraints	Δρ_min_ = −0.38 e Å^−^^3^

### Special details

**Table:**

Geometry. All esds (except the esd in the dihedral angle between two l.s. planes) are estimated using the full covariance matrix. The cell esds are taken into account individually in the estimation of esds in distances, angles and torsion angles; correlations between esds in cell parameters are only used when they are defined by crystal symmetry. An approximate (isotropic) treatment of cell esds is used for estimating esds involving l.s. planes.
Refinement. Refinement of F^2^ against ALL reflections. The weighted R-factor wR and goodness of fit S are based on F^2^, conventional R-factors R are based on F, with F set to zero for negative F^2^. The threshold expression of F^2^ > 2sigma(F^2^) is used only for calculating R-factors(gt) etc. and is not relevant to the choice of reflections for refinement. R-factors based on F^2^ are statistically about twice as large as those based on F, and R- factors based on ALL data will be even larger.

### Fractional atomic coordinates and isotropic or equivalent isotropic displacement parameters (Å^2^)

**Table:**

	*x*	*y*	*z*	*U*_iso_*/*U*_eq_	Occ. (<1)
Cl1	−0.13450 (5)	−0.12001 (5)	0.30389 (4)	0.04416 (14)	
S1	0.38869 (4)	0.27480 (4)	0.39795 (3)	0.02455 (11)	
O1	0.17196 (11)	0.27468 (11)	0.13349 (7)	0.0289 (3)	
O2	0.35241 (13)	0.16152 (11)	0.44212 (9)	0.0352 (3)	
C1	0.26850 (16)	0.25654 (15)	0.28734 (11)	0.0233 (3)	
C2	0.14620 (16)	0.16187 (15)	0.26000 (11)	0.0242 (3)	
C3	0.07727 (16)	0.06820 (15)	0.30474 (12)	0.0275 (4)	
H3	0.1102	0.0547	0.3698	0.033*	
C4	−0.04107 (17)	−0.00427 (16)	0.25041 (12)	0.0306 (4)	
C5	−0.09163 (18)	0.01253 (17)	0.15403 (13)	0.0347 (4)	
H5	−0.1726	−0.0404	0.1191	0.042*	
C6	−0.02470 (18)	0.10514 (17)	0.10986 (12)	0.0329 (4)	
H6	−0.0576	0.1185	0.0447	0.039*	
C7	0.09267 (17)	0.17788 (16)	0.16478 (11)	0.0271 (4)	
C8	0.28051 (16)	0.32102 (15)	0.20894 (11)	0.0253 (4)	
C9	0.37654 (16)	0.42621 (16)	0.18878 (11)	0.0248 (3)	
C10	0.50720 (17)	0.46966 (17)	0.24506 (11)	0.0310 (4)	
H10	0.5374	0.4289	0.2976	0.037*	
C11A	0.59270 (18)	0.57172 (17)	0.22454 (12)	0.0331 (4)	0.9214 (19)
H11	0.6811	0.6004	0.2637	0.040*	0.9214 (19)
C11B	0.59270 (18)	0.57172 (17)	0.22454 (12)	0.0331 (4)	0.08
F1B	0.7030 (8)	0.6258 (10)	0.2897 (7)	0.030 (3)*	0.0786 (19)
C12	0.55272 (18)	0.63305 (17)	0.14834 (12)	0.0338 (4)	
H12	0.6115	0.7034	0.1345	0.041*	
C13A	0.42413 (19)	0.58787 (17)	0.09344 (12)	0.0348 (4)	0.9214 (19)
F1A	0.37983 (13)	0.64640 (12)	0.01946 (9)	0.0534 (4)	0.9214 (19)
C13B	0.42413 (19)	0.58787 (17)	0.09344 (12)	0.0348 (4)	0.08
H13B	0.3951	0.6286	0.0405	0.042*	0.0786 (19)
C14	0.33604 (18)	0.48750 (16)	0.11115 (12)	0.0316 (4)	
H14	0.2481	0.4596	0.0711	0.038*	
C15	0.31917 (18)	0.39296 (15)	0.47604 (11)	0.0293 (4)	
H15A	0.3804	0.4177	0.5405	0.035*	
H15B	0.3233	0.4647	0.4412	0.035*	
C16	0.16734 (19)	0.3549 (2)	0.49948 (13)	0.0426 (5)	
H16A	0.1039	0.3413	0.4370	0.064*	
H16B	0.1396	0.4193	0.5483	0.064*	
H16C	0.1604	0.2796	0.5283	0.064*	
S2	0.40317 (4)	0.78265 (4)	0.38140 (3)	0.02374 (11)	
Cl2	−0.11295 (5)	0.38426 (4)	0.27511 (3)	0.03871 (13)	
O3	0.17390 (12)	0.80626 (11)	0.12789 (8)	0.0301 (3)	
O4	0.37877 (12)	0.66012 (11)	0.41197 (8)	0.0330 (3)	
C17	0.27717 (16)	0.77503 (15)	0.27571 (11)	0.0234 (3)	
C18	0.15482 (16)	0.68113 (15)	0.24602 (11)	0.0249 (3)	
C19	0.09085 (16)	0.58064 (15)	0.28519 (11)	0.0262 (4)	
H19	0.1270	0.5616	0.3475	0.031*	
C20	−0.02740 (17)	0.51006 (16)	0.22934 (12)	0.0288 (4)	
C21	−0.08279 (18)	0.53538 (18)	0.13732 (12)	0.0344 (4)	
H21	−0.1638	0.4836	0.1013	0.041*	
C22	−0.02113 (18)	0.63453 (18)	0.09858 (12)	0.0356 (4)	
H22	−0.0578	0.6535	0.0364	0.043*	
C23	0.09697 (17)	0.70541 (16)	0.15460 (11)	0.0281 (4)	
C24	0.28570 (16)	0.84678 (16)	0.20190 (11)	0.0254 (4)	
C25	0.38173 (16)	0.95328 (15)	0.18337 (11)	0.0244 (3)	
C26	0.50607 (17)	1.00255 (17)	0.24596 (11)	0.0305 (4)	
H26	0.5304	0.9670	0.3032	0.037*	
C27A	0.59358 (18)	1.10291 (17)	0.22457 (12)	0.0321 (4)	0.9214 (19)
H27A	0.6781	1.1352	0.2675	0.039*	0.9214 (19)
C27B	0.59358 (18)	1.10291 (17)	0.22457 (12)	0.0321 (4)	0.08
F2B	0.7032 (9)	1.1542 (11)	0.2920 (7)	0.032 (3)*	0.0786 (19)
C28	0.56134 (18)	1.15759 (16)	0.14231 (12)	0.0322 (4)	
H28	0.6217	1.2268	0.1279	0.039*	
C29A	0.43791 (19)	1.10749 (17)	0.08194 (11)	0.0315 (4)	0.9214 (19)
F2A	0.40153 (13)	1.15850 (12)	0.00136 (9)	0.0489 (4)	0.9214 (19)
C29B	0.43791 (19)	1.10749 (17)	0.08194 (11)	0.0315 (4)	0.08
H29B	0.4137	1.1440	0.0252	0.038*	0.0786 (19)
C30	0.34884 (17)	1.00771 (16)	0.09992 (11)	0.0289 (4)	
H30	0.2653	0.9757	0.0560	0.035*	
C31	0.32775 (18)	0.88245 (16)	0.47356 (11)	0.0300 (4)	
H31A	0.3924	0.9023	0.5367	0.036*	
H31B	0.3216	0.9594	0.4476	0.036*	
C32	0.18165 (19)	0.8292 (2)	0.49831 (13)	0.0429 (5)	
H32A	0.1139	0.8195	0.4384	0.064*	
H32B	0.1515	0.8839	0.5539	0.064*	
H32C	0.1849	0.7496	0.5187	0.064*	

### Atomic displacement parameters (Å^2^)

**Table:**

	*U*^11^	*U*^22^	*U*^33^	*U*^12^	*U*^13^	*U*^23^
Cl1	0.0380 (3)	0.0344 (3)	0.0567 (3)	−0.0087 (2)	−0.0048 (2)	0.0149 (2)
S1	0.0232 (2)	0.0241 (2)	0.02548 (18)	0.00255 (18)	−0.00470 (14)	0.00573 (16)
O1	0.0275 (6)	0.0343 (7)	0.0229 (5)	−0.0012 (5)	−0.0040 (4)	0.0070 (5)
O2	0.0408 (7)	0.0226 (6)	0.0411 (6)	0.0019 (6)	−0.0127 (5)	0.0119 (5)
C1	0.0205 (8)	0.0241 (9)	0.0239 (7)	0.0019 (7)	−0.0016 (6)	0.0025 (6)
C2	0.0215 (8)	0.0243 (9)	0.0255 (7)	0.0042 (7)	0.0005 (6)	0.0007 (7)
C3	0.0264 (8)	0.0269 (9)	0.0290 (7)	0.0054 (8)	−0.0013 (6)	0.0043 (7)
C4	0.0265 (9)	0.0249 (9)	0.0386 (9)	0.0010 (8)	0.0009 (7)	0.0036 (8)
C5	0.0275 (9)	0.0344 (11)	0.0363 (9)	−0.0016 (8)	−0.0065 (7)	−0.0022 (8)
C6	0.0295 (9)	0.0370 (11)	0.0284 (8)	−0.0006 (8)	−0.0056 (7)	0.0028 (8)
C7	0.0247 (8)	0.0291 (9)	0.0256 (7)	0.0010 (8)	−0.0003 (6)	0.0031 (7)
C8	0.0237 (8)	0.0284 (9)	0.0217 (7)	0.0031 (7)	−0.0025 (6)	0.0007 (7)
C9	0.0249 (8)	0.0276 (9)	0.0215 (7)	0.0036 (7)	0.0031 (6)	0.0026 (7)
C10	0.0297 (9)	0.0363 (10)	0.0264 (7)	0.0022 (8)	−0.0006 (6)	0.0078 (7)
C11A	0.0258 (9)	0.0396 (11)	0.0308 (8)	−0.0028 (8)	−0.0004 (7)	0.0051 (8)
C11B	0.0258 (9)	0.0396 (11)	0.0308 (8)	−0.0028 (8)	−0.0004 (7)	0.0051 (8)
C12	0.0333 (9)	0.0322 (10)	0.0345 (8)	−0.0017 (8)	0.0066 (7)	0.0070 (8)
C13A	0.0372 (10)	0.0347 (11)	0.0332 (8)	0.0036 (9)	−0.0002 (7)	0.0110 (8)
F1A	0.0534 (8)	0.0501 (8)	0.0576 (8)	−0.0056 (7)	−0.0120 (6)	0.0330 (7)
C13B	0.0372 (10)	0.0347 (11)	0.0332 (8)	0.0036 (9)	−0.0002 (7)	0.0110 (8)
C14	0.0295 (9)	0.0328 (10)	0.0305 (8)	0.0018 (8)	−0.0049 (7)	0.0057 (8)
C15	0.0364 (9)	0.0222 (9)	0.0272 (7)	0.0036 (8)	−0.0045 (6)	0.0015 (7)
C16	0.0401 (10)	0.0538 (14)	0.0330 (9)	0.0109 (10)	0.0054 (8)	0.0005 (9)
S2	0.0216 (2)	0.0232 (2)	0.02581 (18)	0.00206 (17)	−0.00403 (14)	0.00636 (16)
Cl2	0.0344 (2)	0.0282 (2)	0.0503 (3)	−0.0054 (2)	−0.00282 (19)	0.0093 (2)
O3	0.0279 (6)	0.0360 (7)	0.0233 (5)	−0.0045 (6)	−0.0051 (4)	0.0084 (5)
O4	0.0357 (7)	0.0216 (6)	0.0410 (6)	0.0048 (6)	−0.0089 (5)	0.0084 (5)
C17	0.0208 (8)	0.0243 (9)	0.0233 (7)	0.0010 (7)	−0.0019 (6)	0.0025 (6)
C18	0.0223 (8)	0.0271 (9)	0.0235 (7)	0.0027 (7)	−0.0002 (6)	0.0005 (7)
C19	0.0245 (8)	0.0245 (9)	0.0285 (7)	0.0020 (7)	0.0012 (6)	0.0037 (7)
C20	0.0254 (8)	0.0253 (9)	0.0334 (8)	−0.0005 (8)	0.0026 (6)	0.0018 (7)
C21	0.0279 (9)	0.0379 (11)	0.0314 (8)	−0.0052 (8)	−0.0046 (7)	−0.0002 (8)
C22	0.0317 (9)	0.0431 (12)	0.0276 (8)	−0.0041 (9)	−0.0071 (7)	0.0064 (8)
C23	0.0264 (8)	0.0306 (10)	0.0248 (7)	−0.0022 (8)	−0.0007 (6)	0.0048 (7)
C24	0.0220 (8)	0.0302 (9)	0.0219 (7)	0.0025 (7)	−0.0012 (6)	0.0015 (7)
C25	0.0245 (8)	0.0261 (9)	0.0218 (7)	0.0036 (7)	0.0028 (6)	0.0023 (7)
C26	0.0300 (9)	0.0350 (10)	0.0250 (7)	0.0001 (8)	−0.0014 (6)	0.0069 (7)
C27A	0.0273 (9)	0.0352 (11)	0.0301 (8)	−0.0023 (8)	−0.0027 (7)	0.0030 (8)
C27B	0.0273 (9)	0.0352 (11)	0.0301 (8)	−0.0023 (8)	−0.0027 (7)	0.0030 (8)
C28	0.0331 (9)	0.0281 (10)	0.0347 (8)	−0.0006 (8)	0.0087 (7)	0.0065 (8)
C29A	0.0356 (10)	0.0327 (10)	0.0278 (8)	0.0057 (8)	0.0020 (7)	0.0101 (7)
F2A	0.0538 (8)	0.0487 (8)	0.0449 (6)	−0.0046 (6)	−0.0063 (5)	0.0272 (6)
C29B	0.0356 (10)	0.0327 (10)	0.0278 (8)	0.0057 (8)	0.0020 (7)	0.0101 (7)
C30	0.0285 (9)	0.0313 (10)	0.0261 (7)	0.0028 (8)	−0.0013 (6)	0.0055 (7)
C31	0.0354 (9)	0.0245 (9)	0.0271 (7)	0.0049 (8)	−0.0062 (6)	−0.0011 (7)
C32	0.0381 (10)	0.0522 (13)	0.0365 (9)	0.0083 (10)	0.0069 (8)	−0.0015 (9)

### Geometric parameters (Å, º)

**Table:**

Cl1—C4	1.7403 (18)	S2—O4	1.4882 (12)
S1—O2	1.4909 (12)	S2—C17	1.7818 (14)
S1—C1	1.7818 (14)	S2—C31	1.8018 (16)
S1—C15	1.8003 (16)	S2—O2^i^	3.1361 (11)
S1—O4^i^	3.1898 (11)	Cl2—C20	1.7420 (18)
O1—C7	1.368 (2)	O3—C23	1.366 (2)
O1—C8	1.3827 (17)	O3—C24	1.3828 (17)
C1—C8	1.367 (2)	C17—C24	1.368 (2)
C1—C2	1.445 (2)	C17—C18	1.444 (2)
C2—C7	1.392 (2)	C18—C23	1.394 (2)
C2—C3	1.394 (2)	C18—C19	1.395 (2)
C3—C4	1.382 (2)	C19—C20	1.381 (2)
C3—H3	0.9500	C19—H19	0.9500
C4—C5	1.400 (2)	C20—C21	1.397 (2)
C5—C6	1.373 (3)	C21—C22	1.372 (3)
C5—H5	0.9500	C21—H21	0.9500
C6—C7	1.380 (2)	C22—C23	1.383 (2)
C6—H6	0.9500	C22—H22	0.9500
C8—C9	1.457 (2)	C24—C25	1.460 (2)
C9—C10	1.396 (2)	C25—C30	1.395 (2)
C9—C14	1.398 (2)	C25—C26	1.398 (2)
C10—C11A	1.381 (2)	C26—C27A	1.380 (2)
C10—H10	0.9500	C26—H26	0.9500
C11A—C12	1.382 (2)	C27A—C28	1.382 (2)
C11A—H11	0.9500	C27A—H27A	0.9500
C12—C13A	1.375 (2)	C28—C29A	1.379 (2)
C12—H12	0.9500	C28—H28	0.9500
C13A—F1A	1.3511 (18)	C29A—F2A	1.3483 (17)
C13A—C14	1.366 (2)	C29A—C30	1.367 (2)
C14—H14	0.9500	C30—H30	0.9500
C15—C16	1.517 (2)	C31—C32	1.513 (2)
C15—H15A	0.9900	C31—H31A	0.9900
C15—H15B	0.9900	C31—H31B	0.9900
C16—H16A	0.9800	C32—H32A	0.9800
C16—H16B	0.9800	C32—H32B	0.9800
C16—H16C	0.9800	C32—H32C	0.9800
			
O2—S1—C1	105.42 (7)	O4—S2—C17	105.65 (7)
O2—S1—C15	106.75 (8)	O4—S2—C31	106.46 (8)
C1—S1—C15	99.12 (7)	C17—S2—C31	98.92 (7)
O2—S1—O4^i^	81.93 (5)	O4—S2—O2^i^	83.93 (5)
C1—S1—O4^i^	172.64 (6)	C17—S2—O2^i^	170.27 (6)
C15—S1—O4^i^	78.70 (5)	C31—S2—O2^i^	79.57 (6)
C7—O1—C8	106.76 (12)	C23—O3—C24	106.83 (12)
C8—C1—C2	107.21 (13)	C24—C17—C18	107.22 (13)
C8—C1—S1	127.12 (13)	C24—C17—S2	127.16 (13)
C2—C1—S1	125.44 (12)	C18—C17—S2	125.23 (12)
C7—C2—C3	118.94 (14)	C23—C18—C19	119.15 (14)
C7—C2—C1	104.89 (14)	C23—C18—C17	104.89 (14)
C3—C2—C1	136.17 (14)	C19—C18—C17	135.95 (14)
C4—C3—C2	117.18 (14)	C20—C19—C18	117.03 (14)
C4—C3—H3	121.4	C20—C19—H19	121.5
C2—C3—H3	121.4	C18—C19—H19	121.5
C3—C4—C5	122.81 (16)	C19—C20—C21	122.87 (16)
C3—C4—Cl1	119.26 (13)	C19—C20—Cl2	119.11 (13)
C5—C4—Cl1	117.91 (13)	C21—C20—Cl2	118.02 (13)
C6—C5—C4	120.26 (16)	C22—C21—C20	120.49 (15)
C6—C5—H5	119.9	C22—C21—H21	119.8
C4—C5—H5	119.9	C20—C21—H21	119.8
C5—C6—C7	116.70 (15)	C21—C22—C23	116.67 (15)
C5—C6—H6	121.7	C21—C22—H22	121.7
C7—C6—H6	121.7	C23—C22—H22	121.7
O1—C7—C6	124.97 (14)	O3—C23—C22	125.29 (14)
O1—C7—C2	110.93 (13)	O3—C23—C18	110.92 (14)
C6—C7—C2	124.10 (16)	C22—C23—C18	123.78 (16)
C1—C8—O1	110.19 (14)	C17—C24—O3	110.12 (14)
C1—C8—C9	135.22 (14)	C17—C24—C25	135.92 (14)
O1—C8—C9	114.56 (13)	O3—C24—C25	113.97 (13)
C10—C9—C14	118.43 (16)	C30—C25—C26	118.65 (16)
C10—C9—C8	122.51 (14)	C30—C25—C24	118.94 (14)
C14—C9—C8	119.05 (15)	C26—C25—C24	122.41 (14)
C11A—C10—C9	120.21 (15)	C27A—C26—C25	120.02 (15)
C11A—C10—H10	119.9	C27A—C26—H26	120.0
C9—C10—H10	119.9	C25—C26—H26	120.0
C10—C11A—C12	121.57 (16)	C26—C27A—C28	121.64 (16)
C10—C11A—H11	119.2	C26—C27A—H27A	119.2
C12—C11A—H11	119.2	C28—C27A—H27A	119.2
C13A—C12—C11A	117.11 (17)	C29A—C28—C27A	117.21 (17)
C13A—C12—H12	121.4	C29A—C28—H28	121.4
C11A—C12—H12	121.4	C27A—C28—H28	121.4
F1A—C13A—C14	117.61 (15)	F2A—C29A—C30	117.67 (15)
F1A—C13A—C12	119.02 (17)	F2A—C29A—C28	119.27 (16)
C14—C13A—C12	123.35 (16)	C30—C29A—C28	123.06 (15)
C13A—C14—C9	119.32 (16)	C29A—C30—C25	119.42 (15)
C13A—C14—H14	120.3	C29A—C30—H30	120.3
C9—C14—H14	120.3	C25—C30—H30	120.3
C16—C15—S1	113.45 (12)	C32—C31—S2	113.71 (12)
C16—C15—H15A	108.9	C32—C31—H31A	108.8
S1—C15—H15A	108.9	S2—C31—H31A	108.8
C16—C15—H15B	108.9	C32—C31—H31B	108.8
S1—C15—H15B	108.9	S2—C31—H31B	108.8
H15A—C15—H15B	107.7	H31A—C31—H31B	107.7
C15—C16—H16A	109.5	C31—C32—H32A	109.5
C15—C16—H16B	109.5	C31—C32—H32B	109.5
H16A—C16—H16B	109.5	H32A—C32—H32B	109.5
C15—C16—H16C	109.5	C31—C32—H32C	109.5
H16A—C16—H16C	109.5	H32A—C32—H32C	109.5
H16B—C16—H16C	109.5	H32B—C32—H32C	109.5
			
O2—S1—C1—C8	−163.19 (14)	O4—S2—C17—C24	−156.94 (14)
C15—S1—C1—C8	86.50 (16)	C31—S2—C17—C24	93.07 (15)
O2—S1—C1—C2	10.59 (15)	O4—S2—C17—C18	15.04 (15)
C15—S1—C1—C2	−99.72 (14)	C31—S2—C17—C18	−94.95 (14)
C8—C1—C2—C7	−0.25 (17)	C24—C17—C18—C23	−0.64 (17)
S1—C1—C2—C7	−175.06 (12)	S2—C17—C18—C23	−173.96 (12)
C8—C1—C2—C3	−179.73 (17)	C24—C17—C18—C19	178.21 (17)
S1—C1—C2—C3	5.5 (3)	S2—C17—C18—C19	4.9 (3)
C7—C2—C3—C4	0.4 (2)	C23—C18—C19—C20	0.4 (2)
C1—C2—C3—C4	179.85 (17)	C17—C18—C19—C20	−178.33 (17)
C2—C3—C4—C5	0.6 (2)	C18—C19—C20—C21	0.2 (2)
C2—C3—C4—Cl1	−178.21 (12)	C18—C19—C20—Cl2	−179.33 (12)
C3—C4—C5—C6	−1.0 (3)	C19—C20—C21—C22	−0.7 (3)
Cl1—C4—C5—C6	177.79 (14)	Cl2—C20—C21—C22	178.87 (14)
C4—C5—C6—C7	0.4 (3)	C20—C21—C22—C23	0.5 (3)
C8—O1—C7—C6	−178.81 (16)	C24—O3—C23—C22	−177.52 (16)
C8—O1—C7—C2	1.10 (17)	C24—O3—C23—C18	1.39 (18)
C5—C6—C7—O1	−179.50 (15)	C21—C22—C23—O3	178.95 (16)
C5—C6—C7—C2	0.6 (3)	C21—C22—C23—C18	0.2 (3)
C3—C2—C7—O1	179.06 (13)	C19—C18—C23—O3	−179.56 (13)
C1—C2—C7—O1	−0.53 (17)	C17—C18—C23—O3	−0.47 (18)
C3—C2—C7—C6	−1.0 (2)	C19—C18—C23—C22	−0.6 (3)
C1—C2—C7—C6	179.38 (16)	C17—C18—C23—C22	178.46 (16)
C2—C1—C8—O1	0.93 (18)	C18—C17—C24—O3	1.52 (17)
S1—C1—C8—O1	175.63 (11)	S2—C17—C24—O3	174.67 (11)
C2—C1—C8—C9	178.96 (17)	C18—C17—C24—C25	−178.78 (17)
S1—C1—C8—C9	−6.3 (3)	S2—C17—C24—C25	−5.6 (3)
C7—O1—C8—C1	−1.25 (17)	C23—O3—C24—C17	−1.81 (17)
C7—O1—C8—C9	−179.72 (13)	C23—O3—C24—C25	178.42 (13)
C1—C8—C9—C10	15.5 (3)	C17—C24—C25—C30	−175.60 (17)
O1—C8—C9—C10	−166.56 (14)	O3—C24—C25—C30	4.1 (2)
C1—C8—C9—C14	−163.57 (18)	C17—C24—C25—C26	4.7 (3)
O1—C8—C9—C14	14.4 (2)	O3—C24—C25—C26	−175.58 (14)
C14—C9—C10—C11A	0.7 (2)	C30—C25—C26—C27A	−0.1 (2)
C8—C9—C10—C11A	−178.31 (15)	C24—C25—C26—C27A	179.58 (15)
C9—C10—C11A—C12	−0.3 (3)	C25—C26—C27A—C28	0.4 (3)
C10—C11A—C12—C13A	−0.2 (3)	C26—C27A—C28—C29A	−0.2 (3)
C11A—C12—C13A—F1A	178.81 (16)	C27A—C28—C29A—F2A	179.40 (16)
C11A—C12—C13A—C14	0.3 (3)	C27A—C28—C29A—C30	−0.3 (3)
F1A—C13A—C14—C9	−178.42 (15)	F2A—C29A—C30—C25	−179.12 (14)
C12—C13A—C14—C9	0.1 (3)	C28—C29A—C30—C25	0.6 (3)
C10—C9—C14—C13A	−0.6 (2)	C26—C25—C30—C29A	−0.4 (2)
C8—C9—C14—C13A	178.48 (15)	C24—C25—C30—C29A	179.94 (15)
O2—S1—C15—C16	−46.90 (13)	O4—S2—C31—C32	−45.51 (14)
C1—S1—C15—C16	62.34 (13)	C17—S2—C31—C32	63.82 (14)
O4^i^—S1—C15—C16	−124.80 (12)	O2^i^—S2—C31—C32	−125.93 (13)

Symmetry code: (i) −*x*+1, −*y*+1, −*z*+1.

### Hydrogen-bond geometry (Å, º)

**Table:**

*D*—H···*A*	*D*—H	H···*A*	*D*···*A*	*D*—H···*A*
C15—H15*B*···O4	0.99	2.28	3.233 (2)	160
C31—H31*B*···O2^ii^	0.99	2.26	3.211 (2)	160

Symmetry code: (ii) *x*, *y*+1, *z*.

## References

[bb1] Allen, F. H., Baalham, C. A., Lommerse, J. P. M. & Raithby, P. R. (1998). *Acta Cryst.* B**54**, 320–329.

[bb2] Brandenburg, K. (1998). *DIAMOND* Crystal Impact GbR, Bonn, Germany.

[bb3] Bruker (2009). *APEX2*, *SADABS* and *SAINT* Bruker AXS Inc., Madison, Wisconsin, USA.

[bb4] Choi, H. D., Seo, P. J., Son, B. W. & Lee, U. (2008). *Acta Cryst.* E**64**, o1061. 10.1107/S1600536808013706 PMC2961388 21202580

[bb5] Choi, H. D., Seo, P. J., Son, B. W. & Lee, U. (2010*a*). *Acta Cryst.* E**66**, o402. 10.1107/S1600536810001728 PMC2979967 21579822

[bb6] Choi, H. D., Seo, P. J., Son, B. W. & Lee, U. (2010*b*). *Acta Cryst.* E**66**, o2449. 10.1107/S1600536810033581 PMC3008065 21588770

[bb7] Farrugia, L. J. (2012). *J. Appl. Cryst.* **45**, 849–854.

[bb8] Sheldrick, G. M. (2008). *Acta Cryst.* A**64**, 112–122. 10.1107/S0108767307043930 18156677

